# Fixed-Time Recurrent NN Learning Control of Uncertain Robotic Manipulators with Time-Varying Constraints: Experimental Verification

**DOI:** 10.3390/s23125614

**Published:** 2023-06-15

**Authors:** Qingxin Shi, Changsheng Li, Rui He, Xiaolong Zhu, Xingguang Duan

**Affiliations:** 1 School of Mechatronical Engineering, Beijing Institute of Technology, Beijing 100081, China; 3120185141@bit.edu.cn (Q.S.);; 2 School of Medical Technology, Beijing Institute of Technology, Beijing 100081, China

**Keywords:** recurrent neural network (RNN), barrier Lyapunov function (BLF), fixed-time, manipulators

## Abstract

This paper proposes a learning control framework for the robotic manipulator’s dynamic tracking task demanding fixed-time convergence and constrained output. In contrast with model-dependent methods, the proposed solution deals with unknown manipulator dynamics and external disturbances by virtue of a recurrent neural network (RNN)-based online approximator. First, a time-varying tangent-type barrier Lyapunov function (BLF) is introduced to construct a fixed-time virtual controller. Then, the RNN approximator is embedded in the closed-loop system to compensate for the lumped unknown term in the feedforward loop. Finally, we devise a novel fixed-time, output-constrained neural learning controller by integrating the BLF and RNN approximator into the main framework of the dynamic surface control (DSC). The proposed scheme not only guarantees the tracking errors converge to the small neighborhoods about the origin in a fixed time, but also preserves the actual trajectories always within the prescribed ranges and thus improves the tracking accuracy. Experiment results illustrate the excellent tracking performance and verify the effectiveness of the online RNN estimate for unknown dynamics and external disturbances.

## 1. Introduction

Robotic manipulators are widely used in industrial production, social services, and other fields owing to their unique configurational advantages [1]. However, some control issues cannot be ignored by developers and scholars. For example, the unique configuration complicates dynamics modeling [2]. The friction is affected by speed, temperature, and other factors, and thus is difficult to model accurately [3]. All these issues make high-precision control a challenging task [4]. Many control strategies have been proposed for manipulator motion, such as robust control [5], backstepping control [6], dynamic surface control (DSC) [7], adaptive control [8], and neural network (NN)-based adaptive control [9].

While the adaptive control approach, as an effective control scheme, is useful for various nonlinear dynamic systems, the estimated accuracy for unknown parameters is very limited due to the limitation of simple adaptive laws [10]. To tackle the model uncertainties, intelligent methods such as the NN [9], fuzzy logic theory [11], or Gaussian process regression [12] can be adopted. Studies on the NN show that it has an exceptional ability to mimic continuous nonlinear functions, which is wildly utilized in the field of automatic control [13], system and parameter identification [14], and machine learning [15]. More recently, the combination of NNs and adaptive control has been considered a useful control scheme, proved by fruitful research results which have both higher tracking accuracy and estimated accuracy [16]. Although the NN-based adaptive control has less computational burden than the fuzzy logic-based adaptive control [17], the estimated accuracy of NNs may be reduced by the improper combination of the control framework [18].

According to the propagation mechanism in NNs, NNs can be classified into two groups: feedforward NN (FNN) and recurrent NN (RNN). The radial basis function NN (RBFNN) is the most representative three-layer FNN. RNN is characterized by its capacity to capture, memorize, and reuse dynamic responses through the signal recurrent loops [19]. That is why RNN has received much attention and become a popular approximation approach [14,20]. In the field of tracking control, RNN has been implemented on manipulators and other systems for the dynamic tracking task. One way is online approximation [21]. The other way is offline training [22]. Both ways are effective, and for offline training, it can be adopted as long as the RNN compensation with low-frequency updates can meet our accuracy requirements [12].

For better transient tracking performance, the prescribed performance control (PPC) scheme or the barrier Lyapunov function (BLF) scheme can be incorporated into the controller [23,24]. Although both schemes can achieve the tracking performance of constrained output, it is more difficult to use the PPC for reasonable controller design than the BLF, and it is more likely to lead to design defects from the perspective of stability theory [25]. Typical BLFs include the logarithm-based BLF (Log-BLF) [26] and the tangent-based BLF (Tan-BLF) [27]. Limited by the form of Log-BLF, the Log-BLF is unavailable as the predefined output constraint tends to infinity. However, when compared with the Log-BLF, the Tan-BLF is globally available for any predefined output constraint. Consequently, the Tan-BLF is a more general and practical approach for the control of complex and uncertain systems with or without output constraints [28].

Most of the existing control schemes can only ensure asymptotic stability. From the perspective of practical engineering, it is more valuable to accomplish the control task in the desired time. Compared with asymptotic control, finite-time control can guarantee the errors converge to small neighborhoods about the origin within the finite settling time. However, the settling time of finite-time control is heavily dependent on the initial states of the system. To remove this restriction, the concept of fixed-time control (FTC) has been proposed. FTC is actually a specific situation of finite-time control, whose settling time is bounded and the upper bound of the settling time does not rely on the initial states of the system. For a class of fixed-time backstepping-based control framework, the fixed-time convergence for both virtual controller and real controller needs to be guaranteed [29], and thus it is necessary to consider the design of the fixed-time virtual controller (FTVC) and the fixed-time real controller (FTRC) comprehensively. Moreover, the correct combination of FTC and BLF can better meet the designer’s requirements for control performance. However, considering the form of Tan-BLF, both the FTVC and FTRC should be rigorously and carefully designed, otherwise the undesirable singularity problem will occur [30].

However, in the authors’ opinion, some issues exist in studies that have completed intelligent control for robotic manipulator systems, such as that the design parameters for control signals should be carefully selected to match the real control responsiveness [31] otherwise the control performance may deteriorate; the boundedness of the intermediate error in the RNN learning system should be well addressed. Motivated by the aforementioned issues, for the real-world multi-degree-of-freedom (DoF) manipulator without prior knowledge of dynamics, the dynamic tracking problem is studied using a novel fixed-time, output-constrained RNN learning framework. The stability analysis in theory and the control performance in practice are presented in detail. In contrast to most existing studies and controllers, the distinctive features of the proposed method are given below.

- We propose a controller with the capability of disturbance rejection, uncertainties compensation, as well as constrained output, which satisfies practically fixed-time stable (PFTS).
- An accurate estimate of unknown dynamics and external disturbances is achieved by using an online RNN approximator. A novel RNN dynamics is derived based on Taylor expansion linearization. Furthermore, the RNN dynamics in a robust form are specially constructed to ensure the system stability more reasonably.
- For a class of time-varying BLFs, the Tan-BLF, which is a more general approach than Log-BLF [26] for the control with or without output constraints, is introduced to construct the FTVC, and the corresponding control input term derived from the Tan-BLF is incorporated as a component into the FTRC.

**Figure 1:**
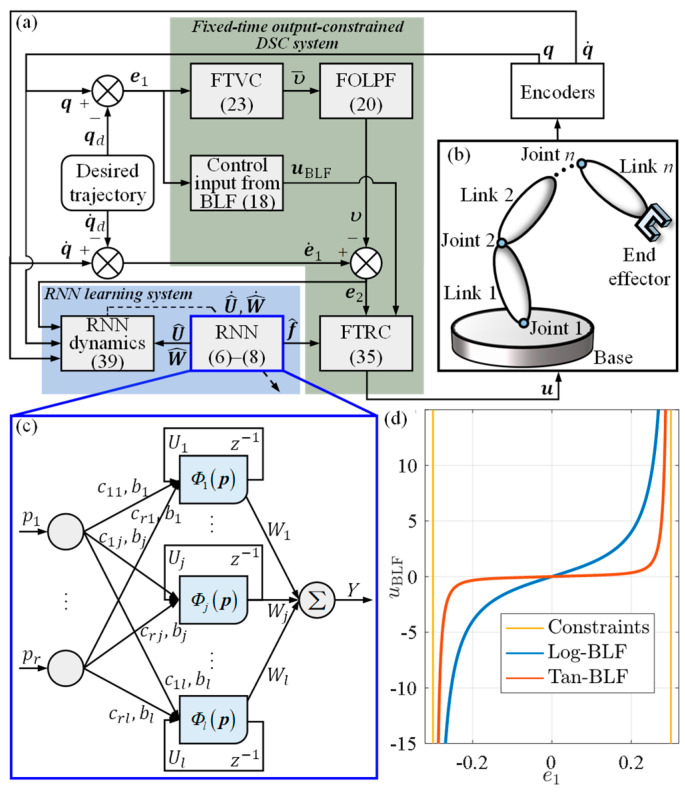
Overall diagram of the fixed-time output-constrained RNN learning control: (**a**) Main control block diagram; (**b**) *n*-DoF serial manipulator; (**c**) Structure of RNN; (**d**) Different types of control input terms derived from BLFs. Take Λ=1 and ρ=0.3, for example.

To the best of our knowledge, there are really limited existing control frameworks that have such performance. The main block diagram for the proposed control framework is illustrated in Figure 1a. The remaining paper is arranged below. Section 2 and Section 3 describe the problem formulation and RNN design, respectively. Section 4 presents the closed-loop control scheme. Section 5 conducts experiments, with conclusions summarized in Section 6.

## 2. Problem Statement and Preliminaries

### 2.1. Notations

Throughout the full text, $\lambda_{max}\left(\cdot \right)$ and $\lambda_{min}\left(\cdot \right)$ refer to the maximum and minimum eigenvalues of a matrix, respectively. ⊙ means Hadamard product. ${\left\lfloor \left.\cdot \right\rceil \right.}^{s}≝{\left|\cdot \right|}^{s}sign\left(\cdot \right)$.

### 2.2. Problem Statement and Formulation

Based on the discussions mentioned above, the objective of this work is to design an FTC for the dynamic tracking of a manipulator system to achieve high-accuracy model-independent control without predefined constraint violations in a theoretically exact fixed time. Specifically, the state tracking errors can converge to the small neighborhoods about the origin and the angle tracking errors will be always confined to the prescribed ranges.

The manipulator dynamics should be modeled first. The serial configuration of an *n*-DoF fixed-base robotic manipulator is depicted in Figure 1b. In the presence of disturbances, the dynamics equation of *n*-DoF manipulator can be described as

(1) $$M\left(q\right)\ddot{q}+C\left(q,\dot{q}\right)\dot{q}+G\left(q\right)=u+\Delta D$$

where $q\in R^{n}$ denotes the joint angle of the *n*-DoF manipulator. $\dot{q}\in R^{n}$ and $\ddot{q}\in R^{n}$ denote the joint angular velocity and acceleration, respectively. $M\left(q\right)\in R^{n\times n}$ denotes the generalized inertia matrix. $C\left(q,\dot{q}\right)\dot{q}\in R^{n}$ stands for Coriolis and the centrifugal forces. $G\left(q\right)\in R^{n}$ denotes the gravitational force matrix. ***u*** $\in R^{n}$ represents the control input. ΔD represents the external disturbances.

### 2.3. Preliminaries

The following mathematical theories and reasonable assumptions are introduced which will be used to prove the correctness of the designed control framework.

**Property 1**  ([32])**.** $\dot{M}\left(q\right)-2C\left(q,\dot{q}\right)$ *is skew symmetric*.

**Property 2**  ([32])**.** $M\left(q\right)$*,* $C\left(q,\dot{q}\right)$*,* *and* $G\left(q\right)$ *are all bounded*.

**Assumption 1.**  *There exists a positive constant* D *such that the external disturbances* ΔD *are bounded with* $\left\|\Delta D\right\|\leq D$.

**Lemma 1**  ([33])**.** *Consider a nonlinear system* $\dot{x}\left(t\right)=f\left(x\left(t\right)\right)$, *where* $x\left(t\right)\in R^{n}$ *and* $f\left(\cdot \right)$ *are used to describe a continuous vector field. Suppose that there exists a positive definite function* $L\left(x\right)$ *such that* $\dot{L}\left(x\right)\leq -\vartheta_{\alpha }L^{\alpha }\left(x\right)-\vartheta_{\beta }L^{\beta }\left(x\right)+E$, *where* $\vartheta_{\alpha }>0$, $\vartheta_{\beta }>0$, 0<α<1, β>1, *and* E>0, *then the nonlinear system is PFTS and* $L\left(x\right)$ *will converge to the following compact set:*

(2) $$\Omega_{L}=\left\{L\left(x\right)\in R|L\left(x\right)\leq min\left\{{\left(\frac{E}{\vartheta_{\alpha }\left(1-\delta \right)}\right)}^{\frac{1}{\alpha }},{\left(\frac{E}{\vartheta_{\beta }\left(1-\delta \right)}\right)}^{\frac{1}{\beta }}\right\}\right\}$$

where 0<δ<1, and thus the fixed settling time $T_{s}$ is bounded by

(3) $$T_{s}\leq \frac{2}{\vartheta_{\alpha }\delta \left(1-\alpha \right)}+\frac{2}{\vartheta_{\beta }\delta \left(\beta -1\right)}$$

**Lemma 2**  ([34])**.** *For any* $x_{i}$, i=1,2,⋯h, 0<α≤1, β>1, *the following inequalities hold:*

(4) $$\left\{\begin{array}{l} {\left(\sum_{i=1}^{h}\left|x_{i}\right|\right)}^{\alpha }\leq \sum_{i=1}^{h}{\left|x_{i}\right|}^{\alpha }\\ {\left(\sum_{i=1}^{h}\left|x_{i}\right|\right)}^{\beta }\leq h^{\beta -1}\sum_{i=1}^{h}{\left|x_{i}\right|}^{\beta }. \end{array}\right.$$

**Lemma 3**  ([34])**.** *For any* $x_{1}$, $x_{2}$, α>0, β>0, σ>0, *the following inequality hold:*

(5) $${\left|x_{1}\right|}^{\alpha }{\left|x_{2}\right|}^{\beta }\leq \frac{\alpha }{\alpha +\beta }\sigma {\left|x_{1}\right|}^{\alpha +\beta }+\frac{\beta }{\alpha +\beta }\sigma^{-\frac{\alpha }{\beta }}{\left|x_{2}\right|}^{\alpha +\beta }$$

## 3. Recurrent Neural Network (RNN) Construction

### 3.1. RNN Design

In pursuit of better tracking performance, a three-layer NN, utilizing the recurrent loops, was specially designed. The structure of the devised RNN is shown in Figure 1c, in which $z^{-1}$ signifies the time delay. Thus, RNN can capture dynamic responses with recurrent loops through $z^{-1}$. Details for the RNN are as follows.

**Layer 1:** Input layer

In the first layer, all needed signals will be first collected, proceeded, and output to the next layer:

(6) $$input:p\;output:X\left(p\right)=p$$

where $p={\left[p_{1},p_{2},\cdots {,p}_{r}\right]}^{T}\in R^{r}$ is the input signal; $X\left(p\right)\in R^{r}$ is the output of Layer 1 and denotes the mapping with respect to the input signal. In this paper, $X\left(p\right)$ is simply selected to be the same as the input signal.

**Layer 2:** Activation layer

Different from the representative RBFNN, the recurrent signals are considered and reused in activation function for RNN:

(7) $$input:X\left(p\right)\;output:\Phi_{j}\left(p\right)=exp\left(-\frac{{\left\|X\left(p\right)+\left({\left(U⊙\Phi \left(t-1\right)\right)}_{j}-c_{j}\right)\times ones\left(r,1\right)\right\|}^{2}}{b^{2}}\right)$$

where $\Phi \left(p\right)={\left[\Phi_{1}\left(p\right),\cdots ,\Phi_{j}\left(p\right),\cdots ,\Phi_{l}\left(p\right)\right]}^{T}\in R^{l}$ is the activation function vector (note that $\Phi \left(p\right)$ is an abbreviated form of $\Phi \left(p,U\right)$ for saving space); $U={\left[U_{1},\cdots ,U_{j},\cdots ,U_{l}\right]}^{T}\in R^{l}$ is the recurrent neural weight vector; $\Phi \left(t-1\right)$ is previous time’s $\Phi \left(p\right)$ obtained by $z^{-1}$; b represents the width of the Gaussian basis function; the center of receptive field $c={\left[c_{1},\cdots ,c_{j},\cdots ,c_{l}\right]}^{T}\in R^{l}$ is evenly spaced according to b.

**Layer 3:** Output layer

Finally, the output of RNN can be obtained using the activation function and forward neural weight:

(8) $$input:\Phi \left(p\right)\;output:Y=W^{T}\Phi \left(p\right)$$

where Y∈R is the final output of RNN; $W={\left[W_{1},\cdots ,W_{j},\cdots ,W_{l}\right]}^{T}\in R^{l}$ is the forward neural weight vector. Note that in this RNN, all neural weights U and W can be tuned based on a desired optimization objective. This completes the construction of RNN.

### 3.2. RNN Approximator

To solve unknown terms in (1), an online RNN approximator is developed. According to the universal approximation theorem [21,35], the RNN approximator is able to mimic a continuous unknown vector field f, which can be expressed as $f=W^{*T}\Phi^{*}\left(p\right)+\varepsilon$, where $\Phi^{*}\left(p\right)$ is the ideal activation function ($\Phi^{*}\left(p\right)$ is an abbreviated form of $\Phi^{*}\left(p,U^{*}\right)$); $U^{*}$ and $W^{*}$ are optimal matrices; the error vector $\varepsilon \in R^{n}$ is bounded with $\left\|\varepsilon \right\|\leq \bar{\varepsilon }$, where $\bar{\varepsilon }$ is a positive constant.

In practice, f can be estimated as $\hat{f}={\hat{W}}^{T}\hat{\Phi }\left(p\right)$, where $\hat{W}$ and $\hat{\Phi }\left(p\right)$ are the estimates of $W^{*}$ and $\Phi^{*}\left(p\right)$, respectively. Note that $\hat{f}$ is the real output of the RNN approximator. To facilitate subsequent mathematical operations of the RNN, some useful formula transformations are given below. The error between f and $\hat{f}$ can be formulated as

(9) $$f-\hat{f}=W^{*T}\Phi^{*}\left(p\right)+\varepsilon -{\hat{W}}^{T}\hat{\Phi }\left(p\right)=-{\tilde{W}}^{T}\hat{\Phi }\left(p\right)-{\hat{W}}^{T}\tilde{\Phi }\left(p\right)+{\tilde{W}}^{T}\tilde{\Phi }\left(p\right)+\varepsilon$$

where $\tilde{W}=\hat{W}-W^{*}$ and $\tilde{\Phi }\left(p\right)=\hat{\Phi }\left(p\right)-\Phi^{*}\left(p\right)$. Taylor expansion linearization is adopted to derive the recurrent neural weight’ dynamics, and $\tilde{\Phi }\left(p\right)$ is converted to the following partially linear form around $\hat{U}$:

(10) $$\tilde{\Phi }\left(p\right)={\left.\tilde{\Phi }\right|}_{U=\hat{U}}+{\left.\frac{\partial \tilde{\Phi }}{\partial U}\right|}_{U=\hat{U}}\left(\hat{U}-U^{*}\right)+\epsilon ={\tilde{\Phi }}_{0}+{\tilde{\Phi }}_{U}\left(p\right)\tilde{U}+\epsilon$$

where ${\tilde{\Phi }}_{0}$ is the first term of Taylor expansion. $\tilde{U}=\hat{U}-U^{*}$ is the estimated error. ϵ is a high-order term. Coefficient matrix ${\tilde{\Phi }}_{U}\left(p\right)\in R^{l\times l}$ is expressed as

(11) $${\tilde{\Phi }}_{U}\left(p\right)=\left[\begin{array}{cc} \begin{array}{cc} \frac{\partial {\tilde{\Phi }}_{1}}{\partial U_{1}}, & \frac{\partial {\tilde{\Phi }}_{1}}{\partial U_{2}},\\ \frac{\partial {\tilde{\Phi }}_{2}}{\partial U_{1}}, & \frac{\partial {\tilde{\Phi }}_{2}}{\partial U_{2}}, \end{array} & \begin{array}{cc} \cdots , & \frac{\partial {\tilde{\Phi }}_{1}}{\partial U_{l}}\\ \cdots & \frac{\partial {\tilde{\Phi }}_{2}}{\partial U_{l}} \end{array}\\ \begin{array}{cc} \vdots & \vdots \\ \frac{\partial {\tilde{\Phi }}_{l}}{\partial U_{1}}, & \frac{\partial {\tilde{\Phi }}_{l}}{\partial U_{2}}, \end{array} & \begin{array}{cc} \ddots & \vdots \\ \cdots & \frac{\partial {\tilde{\Phi }}_{l}}{\partial U_{l}} \end{array} \end{array}\right].$$

Note that ${\tilde{\Phi }}_{U}\left(p\right)$ is available for users, and ${\tilde{\Phi }}_{0}$ is a bounded error vector since $\hat{\Phi }\left(p\right)$ and $\hat{U}$ are bounded and $\Phi^{*}\left(p\right)$ is an ideal constant vector. Therefore, the proposed RNN approximator can be used for approximation calculations.

## 4. Fixed-Time Output-Constrained RNN Learning Control Framework

In this section, a novel fixed-time output-constrained RNN learning controller, designed with the main framework of the DSC, is adopted to solve the dynamic tracking problem of manipulators in the presence of known model dynamics and external disturbances.

### 4.1. Fixed-Time Controller Design

For the tracking control of a second-order manipulator system (1), we consider the following two index errors

(12) $$e_{1}=q-q_{d}$$

(13) $$e_{2}={\dot{e}}_{1}-\upsilon =\dot{q}-{\dot{q}}_{d}-\upsilon$$

where ${\dot{e}}_{1}$ is the time derivative of $e_{1}$; $\upsilon \in R^{n}$ is a filtered virtual controller to be designed later; $q_{d}\in R^{n}$ and ${\dot{q}}_{d}\in R^{n}$ stand for the desired joint angle and angular velocity, respectively. Define the output error constraint as

(14) $$-\rho \left(t\right)<e_{1}\left(t\right)<\rho \left(t\right)$$

where the error constraint $\rho \left(t\right)\in R^{n}$ is predefined as $\rho \left(t\right)={\left[\rho_{1}\left(t\right),\cdots ,\rho_{i}\left(t\right),\cdots \rho_{n}\left(t\right)\right]}^{T}$, where $\rho_{i}\left(t\right)$ is the prescribed time-varying bound expressed as

(15) $$\rho_{i}\left(t\right)=\left(\rho_{i0}-\rho_{i\infty }\right)exp\left(-D_{i}t\right)+\rho_{i\infty }$$

and where $\rho_{i0}$ and $\rho_{i\infty }$ represent the maximum and minimum of $\rho_{i}\left(t\right)$, respectively. It should be noted that $\rho_{i}\left(t\right)$ is restricted and strictly monotonic decreasing to t with $\rho_{i}\left(\infty \right)=\rho_{i\infty }$. $D_{i}$ determines the convergence rate of $\rho_{i}\left(t\right)$. Differentiating error (13) with respect to time and substituting manipulator system (1) into (13) we have

(16) $${\dot{e}}_{2}=M^{-1}\left(u-C\left(q,\dot{q}\right)\dot{q}-G\left(q\right)+\Delta D\right)-{\ddot{q}}_{d}-\dot{\upsilon }$$

where ${\ddot{q}}_{d}\in R^{n}$ stands for the desired angular acceleration; $\dot{\upsilon }$ is the time derivative of ***υ***.

The controller is recursively designed in the following three steps.

**Step 1:** FTVC design

First, construct a time-varying Tan-BLF and the corresponding control input term derived from the Tan-BLF as follows:

(17) $$L_{BLF}=L_{1}=\sum_{i=1}^{n}L_{1i}=\sum_{i=1}^{n}\frac{\Lambda \rho_{i}^{2}\left(t\right)}{\pi }tan\xi_{i},\;\xi_{i}=\frac{\pi e_{1i}^{2}\left(t\right)}{2\rho_{i}^{2}\left(t\right)}$$

(18) $$u_{BLFi}≝\frac{{\partial L}_{BLFi}}{{\partial e}_{1i}}=\frac{\Lambda e_{1i}\left(t\right)}{{cos}^{2}\xi_{i}},i=1,2,\cdots n$$

where Λ is the designed positive constant that determines the maximum of $u_{BLFi}$. Taking the time derivative of the Lyapunov function (17) and substituting (13) into it, we have

(19) $${\dot{L}}_{1}=\sum_{i=1}^{n}\left(\frac{2\Lambda \rho_{i}{\dot{\rho }}_{i}}{\pi }tan\xi_{i}+\frac{\Lambda e_{1i}\left(e_{2i}+\upsilon_{i}-\frac{{\dot{\rho }}_{i}}{\rho_{i}}e_{1i}\right)}{{cos}^{2}\xi_{i}}\right).$$

Considering the form of Tan-BLF, the FTVC cannot be directly used, and the DSC technique is introduced to avoid subsequent complex computations for the time derivative of the FTVC. Design a following first-order low-pass filter (FOLPF)

(20) $$\tau \dot{\upsilon }+\upsilon =\bar{\upsilon },\upsilon \left(0\right)=\bar{\upsilon }\left(0\right)$$

where τ is a small time constant and $\bar{\upsilon }$ denotes the FTVC. Define the filter error as

(21) $$\tilde{\upsilon }=\upsilon -\bar{\upsilon }$$

Differentiating the filter error (21) with respect to time and substituting (20) and (21) into it yields

(22) $$\dot{\tilde{\upsilon }}=\dot{\upsilon }-\dot{\bar{\upsilon }}=-\frac{\tilde{\upsilon }}{\tau }-Q\left(\cdot \right)$$

where $\dot{\overset{-}{\upsilon }}≝Q\left(\cdot \right)$ and $Q\left(\cdot \right)=Q\left(\rho ,\dot{\rho },\ddot{\rho },e_{1},e_{2},\tilde{\upsilon }\right)$ is an unknown continuous vector.

Then, the designed FTVC $\bar{\upsilon }$ is selected as

(23) $$\bar{\upsilon }=\left[\begin{array}{c} \begin{array}{l} \frac{{\dot{\rho }}_{1}e_{1,1}}{\rho_{1}}-\frac{\rho_{1}{\dot{\rho }}_{1}sin\left(2\xi_{1}\right)}{\pi e_{1,1}}-\frac{k_{1}\rho_{1}^{\alpha +1}{sin}^{\frac{\alpha +1}{2}}\xi_{1}}{e_{1,1}{cos}^{\frac{\alpha -3}{2}}\xi_{1}}-\frac{k_{2}\rho_{1}^{\beta +1}{sin}^{\frac{\beta +1}{2}}\xi_{1}}{e_{1,1}{cos}^{\frac{\beta -3}{2}}\xi_{1}}-\frac{e_{1,1}}{2{cos}^{2}\xi_{1}}\\ \frac{{\dot{\rho }}_{2}e_{1,2}}{\rho_{2}}-\frac{\rho_{2}{\dot{\rho }}_{2}sin\left(2\xi_{2}\right)}{\pi e_{1,2}}-\frac{k_{1}\rho_{2}^{\alpha +1}{sin}^{\frac{\alpha +1}{2}}\xi_{2}}{e_{1,2}{cos}^{\frac{\alpha -3}{2}}\xi_{2}}-\frac{k_{2}\rho_{2}^{\beta +1}{sin}^{\frac{\beta +1}{2}}\xi_{2}}{e_{1,2}{cos}^{\frac{\beta -3}{2}}\xi_{2}}-\frac{e_{1,2}}{2{cos}^{2}\xi_{2}} \end{array}\\ \begin{array}{c} \vdots \\ \frac{{\dot{\rho }}_{n}e_{1n}}{\rho_{n}}-\frac{\rho_{n}{\dot{\rho }}_{n}sin\left(2\xi_{n}\right)}{\pi e_{1n}}-\frac{k_{1}\rho_{n}^{\alpha +1}{sin}^{\frac{\alpha +1}{2}}\xi_{n}}{e_{1n}{cos}^{\frac{\alpha -3}{2}}\xi_{n}}-\frac{k_{2}\rho_{n}^{\beta +1}{sin}^{\frac{\beta +1}{2}}\xi_{n}}{e_{1n}{cos}^{\frac{\beta -3}{2}}\xi_{n}}-\frac{e_{1n}}{2{cos}^{2}\xi_{n}} \end{array} \end{array}\right]$$

where $k_{1}>0$, $k_{2}>0$, 0<α<1, and β>1.

**Remark 1.**  *For the FTVC (23), three terms should be discussed:*

*For* $\frac{\rho_{i}{\dot{\rho }}_{i}sin\left(2\xi_{i}\right)}{\pi e_{1i}}$*, when* $e_{1i}\to 0$*, by L’Hospital’s Rule, we can obtain*

(24) $$\underset{e_{1i}\to 0}{\lim}\frac{\rho_{i}{\dot{\rho }}_{i}sin\left(2\xi_{i}\right)}{\pi e_{1i}}=0.$$

*For* $\frac{k_{1}\rho_{i}^{\alpha +1}{sin}^{\frac{\alpha +1}{2}}\xi_{i}}{e_{1i}{cos}^{\frac{\alpha -3}{2}}\xi_{i}}$*, when* $e_{1i}\to 0$ *and* α>0*, it is easy to obtain*

(25) $$\underset{e_{1i}\to 0}{\lim}\frac{k_{1}\rho_{i}^{\alpha +1}{sin}^{\frac{\alpha +1}{2}}\xi_{i}}{e_{1i}{cos}^{\frac{\alpha -3}{2}}\xi_{i}}=0.$$

*Similarly,* $\underset{e_{1i}\to 0}{\lim}\frac{k_{2}\rho_{i}^{\beta +1}{sin}^{\frac{\beta +1}{2}}\xi_{i}}{e_{1i}{cos}^{\frac{\beta -3}{2}}\xi_{i}}=0$ *for any* β>0.

*Thus, the undesirable singularity problem can never occur in the FTVC (23).*

Then, substituting (21) and (23) into (19) yields

(26) $${\dot{L}}_{1}=\sum_{i=1}^{n}\frac{\Lambda e_{1i}e_{2i}}{{cos}^{2}\xi_{i}}-k_{1}\sum_{i=1}^{n}\Lambda {\left(\rho_{i}^{2}tan\xi_{i}\right)}^{\frac{\alpha +1}{2}}-k_{2}\sum_{i=1}^{n}\Lambda {\left(\rho_{i}^{2}tan\xi_{i}\right)}^{\frac{\beta +1}{2}}+\Lambda \sum_{i=1}^{n}\left(\frac{e_{1i}{\tilde{\upsilon }}_{i}}{{cos}^{2}\xi_{i}}-\frac{e_{1i}^{2}}{2{cos}^{4}\xi_{i}}\right)$$

Note that $\Lambda e_{1i}/{cos}^{2}\xi_{i}=u_{BLFi}$. By Young’s inequality, we have

(27) $$\frac{e_{1i}{\tilde{\upsilon }}_{i}}{{cos}^{2}\xi_{i}}-\frac{e_{1i}^{2}}{2{cos}^{4}\xi_{i}}\leq \frac{e_{1i}^{2}}{2{cos}^{4}\xi_{i}}+\frac{{\tilde{\upsilon }}_{i}^{2}}{2}-\frac{e_{1i}^{2}}{2{cos}^{4}\xi_{i}}=\frac{{\tilde{\upsilon }}_{i}^{2}}{2}$$

Substituting (27) into (26) and applying Lemma 2 yields

(28) $${\dot{L}}_{1}\leq \sum_{i=1}^{n}e_{2i}u_{BLFi}-\vartheta_{1}L_{1}^{\frac{\alpha +1}{2}}-\vartheta_{2}L_{1}^{\frac{\beta +1}{2}}+\frac{\Lambda {\tilde{\upsilon }}^{T}\tilde{\upsilon }}{2}$$

where $\vartheta_{1}=k_{1}{\Lambda^{\frac{1-\alpha }{2}}\pi }^{\frac{\alpha +1}{2}}$ and $\vartheta_{2}=k_{2}n^{\frac{1-\beta }{2}}\Lambda^{\frac{1-\beta }{2}}\pi^{\frac{\beta +1}{2}}$.

**Remark 2.**  *For the time-varying* ρ*, when* ρ→∞*, i.e., the output constraint is removed, by L’Hospital’s Rule, we can obtain*

(29) $$L_{1i}=\underset{\rho_{i}\to \infty }{\lim}\frac{\Lambda \rho_{i}^{2}}{\pi }tan\xi_{i}=\frac{\Lambda }{2}e_{1i}^{2}$$

*Thus, the Tan-BLF actually degenerates into the standard quadratic form, which implies that the Tan-BLF is applicable for any* ρ>0*. Then, consider a Log-BLF and its control input term derived from BLF as follows:*

(30) $$L_{i}^{Log}=\frac{1}{2}ln\frac{\rho_{i}^{2}}{\rho_{i}^{2}-e_{1i}^{2}}$$

(31) $$u_{BLFi}^{Log}=\frac{e_{1i}}{\rho_{i}^{2}-e_{1i}^{2}}.$$

*Each type of* $u_{BLF}$ *is shown in* Figure 1*d. It can be observed that two types of* $u_{BLF}$ *have the same trend of change. However, when* ρ→∞*, we have*

(32) $$L_{i}^{Log}=\underset{\rho_{i}\to \infty }{\lim}\frac{1}{2}ln\frac{\rho_{i}^{2}}{\rho_{i}^{2}-e_{1i}^{2}}=0.$$

*Consequently, the Log-BLF (30) becomes unavailable. To sum up, compared with the Log-BLF-based controller, the proposed framework is a more general methodology for controls with or without output constraints.*

**Step 2:** FTRC design

Construct the second Lyapunov function

(33) $$L_{2}=\frac{1}{2}e_{2}^{T}M\left(q\right)e_{2}$$

Taking the time derivative of $L_{2}$, substituting (16) into it, and using Property 1 yields

(34) $${\dot{L}}_{2}=e_{2}^{T}\left(u-M\left(q\right)\left({\ddot{q}}_{d}+\dot{\upsilon }\right)-C\left(q,\dot{q}\right)\left({\dot{q}}_{d}+\upsilon \right)-G\left(q\right)+\Delta D\right).$$

Note that $M\left(q\right)$, $C\left(q,\dot{q}\right)$, $G\left(q\right)$, and ΔD are unknown in advance, and the lumped uncertainties in (34) can be defined as $f≝-M\left(q\right)\left({\ddot{q}}_{d}+\dot{\upsilon }\right)-C\left(q,\dot{q}\right)\left({\dot{q}}_{d}+\upsilon \right)-G\left(q\right)+\Delta D$. To deal with f, the RNN approximator is utilized and embedded in the controller, i.e., $\hat{f}={\hat{W}}^{T}{\hat{W}}^{T}\hat{\Phi }\left(p\right)$ with $p={\left[q^{T},{\dot{q}}^{T},\upsilon^{T}\right]}^{T}$. Accordingly, the RNN-based FTRC is designed as

(35) $$u=-\left[\begin{array}{c} \begin{array}{c} u_{BLF1}\\ u_{BLF2} \end{array}\\ \begin{array}{c} \vdots \\ u_{BLFn} \end{array} \end{array}\right]-K_{1}{\left\lfloor \left.e_{2}\right\rceil \right.}^{\alpha }-K_{2}{\left\lfloor \left.e_{2}\right\rceil \right.}^{\beta }-\frac{1}{2}e_{2}-{\hat{W}}^{T}\hat{\Phi }\left(p\right)$$

where $K_{1}>0$ and $K_{2}>0$.

Substituting FTRC (35) and error (9) into (34) yields

(36) $${\dot{L}}_{2}=-\sum_{i=1}^{n}e_{2i}u_{BLFi}-e_{2}^{T}\left(K_{1}{\left\lfloor \left.e_{2}\right\rceil \right.}^{\alpha }+K_{2}{\left\lfloor \left.e_{2}\right\rceil \right.}^{\beta }\right)-\frac{1}{2}e_{2}^{T}e_{2}+e_{2}^{T}\left(W^{*T}\Phi^{*}\left(p\right)+\varepsilon -{\hat{W}}^{T}\hat{\Phi }\left(p\right)\right)\;=-\sum_{i=1}^{n}e_{2i}u_{BLFi}-e_{2}^{T}\left(K_{1}{\left\lfloor \left.e_{2}\right\rceil \right.}^{\alpha }+K_{2}{\left\lfloor \left.e_{2}\right\rceil \right.}^{\beta }\right)-\frac{1}{2}e_{2}^{T}e_{2}+e_{2}^{T}\left(-{\tilde{W}}^{T}\hat{\Phi }\left(p\right)-{\hat{W}}^{T}{\tilde{\Phi }}_{U}\left(p\right)\tilde{U}-{\hat{W}}^{T}\left({\tilde{\Phi }}_{0}+\epsilon \right)+{\tilde{W}}^{T}\tilde{\Phi }\left(p\right)+\varepsilon \right)\;=-\sum_{i=1}^{n}e_{2i}u_{BLFi}-K_{1}{\left(e_{2}^{T}e_{2}\right)}^{\frac{\alpha +1}{2}}-K_{2}{\left(e_{2}^{T}e_{2}\right)}^{\frac{\beta +1}{2}}+e_{2}^{T}\left(-{\tilde{W}}^{T}\hat{\Phi }\left(p\right)-{\hat{W}}^{T}{\tilde{\Phi }}_{U}\left(p\right)\tilde{U}\right)-\frac{1}{2}e_{2}^{T}e_{2}+e_{2}^{T}\Delta$$

where $\Delta =-{\hat{W}}^{T}\left({\tilde{\Phi }}_{0}+\epsilon \right)+{\tilde{W}}^{T}\tilde{\Phi }\left(p\right)+\varepsilon$. Note that Δ is bounded satisfying $\left\|\Delta \right\|\leq \bar{\Delta }$, where $\bar{\Delta }$ is a positive constant. Thus, we have the following inequality:

(37) $$e_{2}^{T}\Delta \leq \frac{1}{2}e_{2}^{T}e_{2}+\frac{1}{2}{\left\|\Delta \right\|}^{2}\leq \frac{1}{2}e_{2}^{T}e_{2}+\frac{1}{2}{\bar{\Delta }}^{2}$$

Substituting (37) into (36), we further have

(38) $${\dot{L}}_{2}\leq -\sum_{i=1}^{n}e_{2i}u_{BLFi}-K_{1}{\left(e_{2}^{T}e_{2}\right)}^{\frac{\alpha +1}{2}}-K_{2}{\left(e_{2}^{T}e_{2}\right)}^{\frac{\beta +1}{2}}+e_{2}^{T}\left(-{\tilde{W}}^{T}\hat{\Phi }\left(p\right)-{\hat{W}}^{T}{\tilde{\Phi }}_{U}\left(p\right)\tilde{U}\right)-\frac{1}{2}e_{2}^{T}e_{2}+\frac{1}{2}e_{2}^{T}e_{2}+\frac{1}{2}{\bar{\Delta }}^{2}\;=-\sum_{i=1}^{n}e_{2i}u_{BLFi}-\vartheta_{3}L_{2}^{\frac{\alpha +1}{2}}-\vartheta_{4}L_{2}^{\frac{\beta +1}{2}}+e_{2}^{T}\left(-{\tilde{W}}^{T}\hat{\Phi }\left(p\right)-{\hat{W}}^{T}{\tilde{\Phi }}_{U}\left(p\right)\tilde{U}\right)+\frac{1}{2}{\bar{\Delta }}^{2}$$

where $\vartheta_{3}=\frac{K_{1}2^{\frac{\alpha +1}{2}}}{\lambda_{max}^{\frac{\alpha +1}{2}}\left(M\right)}$ and $\vartheta_{4}=\frac{K_{2}2^{\frac{\beta +1}{2}}}{\lambda_{max}^{\frac{\beta +1}{2}}\left(M\right)}$.

**Step 3:** Online RNN learning design

The weights of RNN are designed to be online-tuned based on the RNN dynamics derived from the Lyapunov theory. Then, the RNN dynamics in a robust form for the RNN-based FTRC (35) are designed as

(39) $$\left\{\begin{array}{l} {\dot{\hat{U}}}_{i}=\gamma_{1i}{\tilde{\Phi }}_{Ui}\left(p_{i}\right){\hat{W}}_{i}e_{2i}-\eta_{1i}\gamma_{1i}{\hat{U}}_{i}\\ {\dot{\hat{W}}}_{i}=\gamma_{2i}{\hat{\Phi }}_{i}\left(p_{i}\right)e_{2i}-\eta_{2i}\gamma_{2i}{\hat{W}}_{i} \end{array},\right.i=1,2,\cdots n$$

where $\gamma_{1i}\in R^{l\times l}$ and $\gamma_{2i}\in R^{l\times l}$ are diagonal positive definite matrices; $\eta_{1i}$ and $\eta_{2i}$ are small positive constants, and then construct the third Lyapunov function as

(40) $$L_{3}=\frac{1}{2}\sum_{i=1}^{n}{\tilde{U}}_{i}^{T}\gamma_{1i}^{-1}{\tilde{U}}_{i}.$$

Differentiating $L_{3}$ with respect to time and substituting the RNN dynamics (39) into it, we have

(41) $${\dot{L}}_{3}=\sum_{i=1}^{n}e_{2i}{\hat{W}}_{i}^{T}{\tilde{\Phi }}_{Ui}\left(p_{i}\right){\tilde{U}}_{i}-\sum_{i=1}^{n}\eta_{1i}{\tilde{U}}_{i}^{T}{\hat{U}}_{i}.$$

Note that $-{\tilde{U}}_{i}^{T}{\hat{U}}_{i}\leq -\frac{1}{2}{\left\|{\tilde{U}}_{i}\right\|}^{2}+\frac{1}{2}{\left\|U_{i}^{*}\right\|}^{2}$, and then substituting this inequality into (41) yields

(42) $${\dot{L}}_{3}\leq \sum_{i=1}^{n}e_{2i}{\hat{W}}_{i}^{T}{\tilde{\Phi }}_{Ui}\left(p_{i}\right){\tilde{U}}_{i}-\sum_{i=1}^{n}\frac{\eta_{1i}}{2}{\left\|{\tilde{U}}_{i}\right\|}^{2}+\sum_{i=1}^{n}\frac{\eta_{1i}}{2}{\left\|U_{i}^{*}\right\|}^{2}\;=\sum_{i=1}^{n}e_{2i}{\hat{W}}_{i}^{T}{\tilde{\Phi }}_{Ui}\left(p_{i}\right){\tilde{U}}_{i}-\sum_{i=1}^{n}{\left(\frac{\eta_{1i}}{4}{\left\|{\tilde{U}}_{i}\right\|}^{2}\right)}^{\frac{\alpha +1}{2}}-\sum_{i=1}^{n}{\left(\frac{\eta_{1i}}{4}{\left\|{\tilde{U}}_{i}\right\|}^{2}\right)}^{\frac{\beta +1}{2}}+\Theta_{1}+\sum_{i=1}^{n}\frac{\eta_{1i}}{2}{\left\|U_{i}^{*}\right\|}^{2}$$

where $\Theta_{1}=\sum_{i=1}^{n}{\left(\frac{\eta_{1i}}{4}{\left\|{\tilde{U}}_{i}\right\|}^{2}\right)}^{\frac{\alpha +1}{2}}+\sum_{i=1}^{n}{\left(\frac{\eta_{1i}}{4}{\left\|{\tilde{U}}_{i}\right\|}^{2}\right)}^{\frac{\beta +1}{2}}-\sum_{i=1}^{n}\frac{\eta_{1i}}{2}{\left\|{\tilde{U}}_{i}\right\|}^{2}$. Then, for $\Theta_{1}$, the following two cases should be considered:

(1) If $\frac{\eta_{1i}}{4}{\left\|{\tilde{U}}_{i}\right\|}^{2}\geq 1$, we have
  (43) $${\left(\frac{\eta_{1i}}{4}{\left\|{\tilde{U}}_{i}\right\|}^{2}\right)}^{\frac{\alpha +1}{2}}+{\left(\frac{\eta_{1i}}{4}{\left\|{\tilde{U}}_{i}\right\|}^{2}\right)}^{\frac{\beta +1}{2}}-\frac{\eta_{1i}}{2}{\left\|{\tilde{U}}_{i}\right\|}^{2}\leq {\left(\frac{\eta_{1i}}{4}{\left\|{\tilde{U}}_{i}\right\|}^{2}\right)}^{\frac{\beta +1}{2}}-\frac{\eta_{1i}}{4}{\left\|{\tilde{U}}_{i}\right\|}^{2}.$$

(2) If $\frac{\eta_{1i}}{4}{\left\|{\tilde{U}}_{i}\right\|}^{2}<1$, applying Lemma 3, we can obtain
  (44) $${\left(\frac{\eta_{1i}}{4}{\left\|{\tilde{U}}_{i}\right\|}^{2}\right)}^{\frac{\alpha +1}{2}}+{\left(\frac{\eta_{1i}}{4}{\left\|{\tilde{U}}_{i}\right\|}^{2}\right)}^{\frac{\beta +1}{2}}-\frac{\eta_{1i}}{2}{\left\|{\tilde{U}}_{i}\right\|}^{2}\leq {\left(\frac{\eta_{1i}}{4}{\left\|{\tilde{U}}_{i}\right\|}^{2}\right)}^{\frac{\alpha +1}{2}}-\frac{\eta_{1i}}{4}{\left\|{\tilde{U}}_{i}\right\|}^{2}\leq \left(1-\bar{\alpha }\right){\bar{\alpha }}^{\frac{\bar{\alpha }}{1-\bar{\alpha }}}$$
  where $\bar{\alpha }=\frac{\alpha +1}{2}$. Suppose that there exist unknown constants $d_{1i}\left(i=1,2,\cdots n\right)$ and compact sets $\Omega_{A_{i}}$ such that $\Omega_{A_{i}}=\left\{{\tilde{U}}_{i}\in R^{l}|\left\|{\tilde{U}}_{i}\right\|\leq d_{1i}\right\}$, i=1,2,⋯n. Based on inequalities (43) and (44), we have
  (45) $${\left(\frac{\eta_{1i}}{4}{\left\|{\tilde{U}}_{i}\right\|}^{2}\right)}^{\frac{\alpha +1}{2}}+{\left(\frac{\eta_{1i}}{4}{\left\|{\tilde{U}}_{i}\right\|}^{2}\right)}^{\frac{\beta +1}{2}}-\frac{\eta_{1i}}{2}{\left\|{\tilde{U}}_{i}\right\|}^{2}\leq \Xi_{1i}$$
  where $\Xi_{1i}$ is defined as
  (46) $$\Xi_{1i}=\left\{\begin{array}{l} \left(1-\bar{\alpha }\right){\bar{\alpha }}^{\frac{\bar{\alpha }}{1-\bar{\alpha }}},d_{1i}<\frac{2}{\sqrt{\eta_{1i}}}\\ {\left(\frac{\eta_{1i}}{4}d_{1i}^{2}\right)}^{\frac{\beta +1}{2}}-\frac{\eta_{1i}}{4}d_{1i}^{2},d_{1i}\geq \frac{2}{\sqrt{\eta_{1i}}}. \end{array}\right.$$
  Substituting (45) into (42) and applying Lemma 2, we have
  (47) $${\dot{L}}_{3}\leq \sum_{i=1}^{n}e_{2i}{\hat{W}}_{i}^{T}{\tilde{\Phi }}_{Ui}\left(p_{i}\right){\tilde{U}}_{i}-\vartheta_{5}L_{3}^{\frac{\alpha +1}{2}}-\vartheta_{6}L_{3}^{\frac{\beta +1}{2}}+\sum_{i=1}^{n}\left(\Xi_{1i}+\frac{\eta_{1i}}{2}{\left\|U_{i}^{*}\right\|}^{2}\right)$$
  where $\vartheta_{5}=\frac{{\eta_{1i}}^{\frac{\alpha +1}{2}}}{2^{\frac{\alpha +1}{2}}\lambda_{max}^{\frac{\alpha +1}{2}}\left(\gamma_{1i}^{-1}\right)}$ and $\vartheta_{6}=\frac{n^{\frac{1-\beta }{2}}{\eta_{1i}}^{\frac{\beta +1}{2}}}{2^{\frac{\beta +1}{2}}\lambda_{max}^{\frac{\beta +1}{2}}\left(\gamma_{1i}^{-1}\right)}$.

Similarly, construct the fourth Lyapunov function

(48) $$L_{4}=\frac{1}{2}\sum_{i=1}^{n}{\tilde{W}}_{i}^{T}\gamma_{2i}^{-1}{\tilde{W}}_{i}$$

and then the time derivative of $L_{4}$ has the following similar form to ${\dot{L}}_{3}$:

(49) $${\dot{L}}_{4}\leq \sum_{i=1}^{n}e_{2i}{\tilde{W}}_{i}^{T}{\hat{\Phi }}_{i}\left(p_{i}\right)-\vartheta_{7}L_{4}^{\frac{\alpha +1}{2}}-\vartheta_{8}L_{4}^{\frac{\beta +1}{2}}+\sum_{i=1}^{n}\left(\Xi_{2i}+\frac{\eta_{2i}}{2}{\left\|W_{i}^{*}\right\|}^{2}\right)$$

where $\vartheta_{7}=\frac{{\eta_{2i}}^{\frac{\alpha +1}{2}}}{2^{\frac{\alpha +1}{2}}\lambda_{max}^{\frac{\alpha +1}{2}}\left(\gamma_{2i}^{-1}\right)}$ and $\vartheta_{8}=\frac{n^{\frac{1-\beta }{2}}{\eta_{2i}}^{\frac{\beta +1}{2}}}{2^{\frac{\beta +1}{2}}\lambda_{max}^{\frac{\beta +1}{2}}\left(\gamma_{2i}^{-1}\right)}$.

### 4.2. Stability Analysis for Closed-Loop System

After the above subsystems’ design and analysis, we propose Theorem 1 for the devised main control framework.

**Theorem 1.**  *Consider the manipulator system (1) under Assumption 1 together with the FTVC (23), FTRC(35), FOLPF (20), and RNN dynamics (39), and then the closed-loop system is PFTS. Moreover, signals* $e_{1}$*,* $\tilde{\upsilon }$*,* $e_{2}$*,* ${\tilde{U}}_{i}$*, and* ${\tilde{W}}_{i}$*remain in the following compact sets within fixed time:*

(50) $$\left\{\begin{array}{l} \begin{array}{l} \Omega_{e_{1}}=\left\{e_{1}\in R^{n}|\left|e_{1i}\right|<\rho_{i}\right\}\\ \begin{array}{l} \Omega_{\tilde{\upsilon }}=\left\{\tilde{\upsilon }\in R^{n}|\left\|\tilde{\upsilon }\right\|\leq \sqrt{2G}\right\}\\ \Omega_{e_{2}}=\left\{e_{2}\in R^{n}|\left\|e_{2}\right\|\leq \sqrt{\frac{2G}{\lambda_{min}\left(M\right)}}\right\} \end{array} \end{array}\\ \begin{array}{l} \Omega_{{\tilde{U}}_{i}}=\left\{{\tilde{U}}_{i}\in R^{l}|\left\|{\tilde{U}}_{i}\right\|\leq \sqrt{\frac{2G}{\lambda_{min}\left(\gamma_{1i}^{-1}\right)}}\right\}\\ \Omega_{{\tilde{W}}_{i}}=\left\{{\tilde{W}}_{i}\in R^{l}|\left\|{\tilde{W}}_{i}\right\|\leq \sqrt{\frac{2G}{\lambda_{min}\left(\gamma_{2i}^{-1}\right)}}\right\} \end{array} \end{array}\right.,i=1,2,\cdots n$$

*where* G *is a positive constant defined in the sequel.*

**Proof.**  Construct the following final Lyapunov function:

(51) $$L=\sum_{m=1}^{5}L_{m}$$

where $L_{5}=\frac{1}{2}{\tilde{\upsilon }}^{T}\tilde{\upsilon }$. Differentiating $L_{5}$ with respect to time along (22) yields

(52) $${\dot{L}}_{5}={\tilde{\upsilon }}^{T}\left(\dot{\upsilon }-\dot{\overset{-}{\upsilon }}\right)=-\frac{{\tilde{\upsilon }}^{T}\tilde{\upsilon }}{\tau }-{\tilde{\upsilon }}^{T}Q\left(\cdot \right)$$

Consider the following compact sets:

(53) $$\left\{\begin{array}{l} \Omega_{\rho }=\left\{\left(\rho ,\dot{\rho },\ddot{\rho }\right)|{\left\|\rho \right\|}^{2}+{\left\|\dot{\rho }\right\|}^{2}+{\left\|\ddot{\rho }\right\|}^{2}\leq d_{3}\right\}\\ \Omega_{ℵ}=\left\{\left(e_{1},e_{2},\tilde{\upsilon }\right)|{\left\|e_{1}\right\|}^{2}+{\left\|e_{2}\right\|}^{2}+{\left\|\tilde{\upsilon }\right\|}^{2}\leq d_{4}\right\} \end{array}\right.$$

where $d_{3}$ and $d_{4}$ are positive constants. It follows that $\Omega =\Omega_{\rho }\times \Omega_{ℵ}$ is also a compact set. From (22) all of the error variables in $Q\left(\cdot \right)$ are bounded in the compact set Ω, which means that a positive constant Q exists with $\left\|Q\left(\cdot \right)\right\|\leq Q$, and then using Young’s inequality we can obtain

(54) $$-{\tilde{\upsilon }}^{T}Q\left(\cdot \right)\leq \frac{\omega {\tilde{\upsilon }}^{T}\tilde{\upsilon }}{2}+\frac{Q^{2}}{2\omega }$$

where ω is a designed positive constant. Combining (52) and (54) yields

(55) $${\dot{L}}_{5}\leq -\left(\frac{1}{\tau }-\frac{\omega }{2}\right){\tilde{\upsilon }}^{T}\tilde{\upsilon }+\frac{Q^{2}}{2\omega }.$$

Finally, taking the time derivative of L along (28), (38), (47), (49), and (55) yields

(56) $$\dot{L}=\sum_{m=1}^{5}{\dot{L}}_{m}\leq -\vartheta_{1}L_{1}^{\frac{\alpha +1}{2}}-\vartheta_{2}L_{1}^{\frac{\beta +1}{2}}-\vartheta_{3}L_{2}^{\frac{\alpha +1}{2}}-\vartheta_{4}L_{2}^{\frac{\beta +1}{2}}-\vartheta_{5}L_{3}^{\frac{\alpha +1}{2}}-\vartheta_{6}L_{3}^{\frac{\beta +1}{2}}-\vartheta_{7}L_{4}^{\frac{\alpha +1}{2}}-\vartheta_{8}L_{4}^{\frac{\beta +1}{2}}-{\left(\frac{N}{2}{\tilde{\upsilon }}^{T}\tilde{\upsilon }\right)}^{\frac{\alpha +1}{2}}\;-{\left(\frac{N}{2}{\tilde{\upsilon }}^{T}\tilde{\upsilon }\right)}^{\frac{\beta +1}{2}}+\Theta_{3}+\frac{1}{2}{\bar{\Delta }}^{2}+\frac{Q^{2}}{2\omega }+\sum_{i=1}^{n}\left(\Xi_{1i}+\frac{\eta_{1i}}{2}{\left\|U_{i}^{*}\right\|}^{2}{+\Xi }_{2i}+\frac{\eta_{2i}}{2}{\left\|W_{i}^{*}\right\|}^{2}\right)$$

where $N=\left(\frac{1}{\tau }-\frac{\omega }{2}-\frac{\Lambda }{2}\right)$ and $\Theta_{3}={\left(\frac{N}{2}{\tilde{\upsilon }}^{T}\tilde{\upsilon }\right)}^{\frac{\alpha +1}{2}}+{\left(\frac{N}{2}{\tilde{\upsilon }}^{T}\tilde{\upsilon }\right)}^{\frac{\beta +1}{2}}-N{\tilde{\upsilon }}^{T}\tilde{\upsilon }$. Note that by choosing the appropriate parameters we can guarantee N>0. The discussions for $\Theta_{3}$ are similar to those for $\Theta_{1}$, and the following inequality therefore holds:

(57) $$\Theta_{3}={\left(\frac{N}{2}{\tilde{\upsilon }}^{T}\tilde{\upsilon }\right)}^{\frac{\alpha +1}{2}}+{\left(\frac{N}{2}{\tilde{\upsilon }}^{T}\tilde{\upsilon }\right)}^{\frac{\beta +1}{2}}-N{\tilde{\upsilon }}^{T}\tilde{\upsilon }\leq \Xi_{3}.$$

Substituting (57) into (56) yields

(58) $$\dot{L}\leq -\vartheta_{1}L_{1}^{\frac{\alpha +1}{2}}-\vartheta_{2}L_{1}^{\frac{\beta +1}{2}}-\vartheta_{3}L_{2}^{\frac{\alpha +1}{2}}-\vartheta_{4}L_{2}^{\frac{\beta +1}{2}}-\vartheta_{5}L_{3}^{\frac{\alpha +1}{2}}-\vartheta_{6}L_{3}^{\frac{\beta +1}{2}}-\vartheta_{7}L_{4}^{\frac{\alpha +1}{2}}-\vartheta_{8}L_{4}^{\frac{\beta +1}{2}}-\vartheta_{9}L_{5}^{\frac{\alpha +1}{2}}-\vartheta_{10}L_{5}^{\frac{\beta +1}{2}}+\frac{1}{2}{\bar{\Delta }}^{2}\;+\frac{Q^{2}}{2\omega }+\Xi_{3}+\sum_{i=1}^{n}\left(\Xi_{1i}+\Xi_{2i}+\frac{\eta_{1i}}{2}{\left\|U_{i}^{*}\right\|}^{2}+\frac{\eta_{2i}}{2}{\left\|W_{i}^{*}\right\|}^{2}\right)$$

where $\vartheta_{9}=N^{\frac{\alpha +1}{2}}$ and $\vartheta_{10}=N^{\frac{\beta +1}{2}}$.

Applying Lemma 2 to (58), we have

(59) $$\dot{L}\leq -\vartheta_{\alpha }L^{\frac{\alpha +1}{2}}-\vartheta_{\beta }L^{\frac{\beta +1}{2}}+E$$

where $\vartheta_{\alpha }=min\left\{\vartheta_{1},\vartheta_{3},\vartheta_{5},\vartheta_{7},\vartheta_{9}\right\}$, $\vartheta_{\beta }=5^{\frac{1-\beta }{2}}min\left\{\vartheta_{2},\vartheta_{4},\vartheta_{6},\vartheta_{8},\vartheta_{10}\right\}$, and $E=\frac{1}{2}{\bar{\Delta }}^{2}+\frac{Q^{2}}{2\omega }+\Xi_{3}+\sum_{i=1}^{n}\left(\Xi_{1i}+\Xi_{2i}+\frac{\eta_{1i}}{2}{\left\|U_{i}^{*}\right\|}^{2}+\frac{\eta_{2i}}{2}{\left\|W_{i}^{*}\right\|}^{2}\right)$.

Finally, by Lemma 1, L will converge to the following compact set:

(60) $$\Omega_{L}=\left\{L\left(x\right)\in R|L\left(x\right)\leq min\left\{{\left(\frac{E}{\vartheta_{\alpha }\left(1-\delta \right)}\right)}^{\frac{2}{\alpha +1}},{\left(\frac{E}{\vartheta_{\beta }\left(1-\delta \right)}\right)}^{\frac{2}{\beta +1}}\right\}\right\}$$

where 0<δ<1. The fixed settling time $T_{s}$ is bounded as

(61) $$T_{s}\leq \frac{2}{\vartheta_{\alpha }\delta \left(1-\frac{\alpha +1}{2}\right)}+\frac{2}{\vartheta_{\beta }\delta \left(\frac{\beta +1}{2}-1\right)}$$

Define the mentioned positive constant $G=min\left\{{\left(\frac{E}{\vartheta_{\alpha }\left(1-\delta \right)}\right)}^{\frac{2}{\alpha +1}},{\left(\frac{E}{\vartheta_{\beta }\left(1-\delta \right)}\right)}^{\frac{2}{\beta +1}}\right\}$. Using (51), we have

(62) $$\frac{\Lambda \rho_{i}^{2}}{\pi }tan\xi_{i}\leq G,i=1,2,\cdots n.$$

Using the maximum of arctan function and rearranging (62) yields

(63) $$\frac{\pi e_{1i}^{2}}{2\rho_{i}^{2}}\leq atan\left(\frac{\pi G}{\Lambda \rho_{i}^{2}}\right)<\frac{\pi }{2},i=1,2,\cdots n$$

i.e., $\left|e_{1i}\right|<\rho_{i}$. Thus, the closed-loop signal $e_{1}$ remains in the compact set $\Omega_{e_{1}}$. Likewise, the closed-loop signals $\tilde{\upsilon }$, $e_{2}$, ${\tilde{U}}_{i}$, and ${\tilde{W}}_{i}$ can converge to the compact sets $\Omega_{\tilde{\upsilon }}$, $\Omega_{e_{2}}$, $\Omega_{{\tilde{U}}_{i}}$, and $\Omega_{{\tilde{W}}_{i}}$ defined as (50) within the fixed time, respectively. Furthermore, the tracking errors can never exceed the prescribed time-varying bounds, i.e., $-\rho \left(t\right)<e_{1}\left(t\right)<\rho \left(t\right)$, provided that $-\rho \left(0\right)<e_{1}\left(0\right)<\rho \left(0\right)$. This completes the proof of Theorem 1. □

**Remark 3.**  *The control performance indicators involved in this paper mainly include system stability, settling time, and tracking error. To realize the fixed-time stability for the closed-loop system, the fixed-time stability criteria should be satisfied, namely, the Lyapunov function* $L\left(x\right)>0$ *and* $\dot{L}\left(x\right)\leq -\vartheta_{\alpha }L^{\alpha }\left(x\right)-\vartheta_{\beta }L^{\beta }\left(x\right)+E$*. In this case, the settling time satisfies* $T_{s}\leq \frac{2}{\vartheta_{\alpha }\delta \left(1-\frac{\alpha +1}{2}\right)}+\frac{2}{\vartheta_{\beta }\delta \left(\frac{\beta +1}{2}-1\right)}$*. It is clear that the boundary of the settling time is not affected by initial conditions. Different settling time ranges can be adjusted prior based on the practical performance requirements.*

**Remark 4.**  *The control performance of the closed-loop system depends on the following adjustable design parameters: (1) It can be seen from the stability analysis that parameters* $k_{1}$*,* $k_{2}$*,* $K_{1}$*, and* $K_{2}$ *can adjust the convergence error and convergence accuracy at the same time. By selecting larger* $k_{2}$ *and* $K_{2}$*, and appropriate* $k_{1}$ *and* $K_{1}$*, the convergence speed can be improved and the final error can be reduced. (2) The exponents* α *and* β *determine the boundary of the convergence time and influence the convergence accuracy. Choosing suitable exponents can reduce the convergence time and improve the convergence accuracy. (3) If* $\gamma_{1i}$ *and* $\gamma_{2i}$ *are selected too small, the RNN estimate is not accurate enough. If they are selected too large, the overshoot of the system becomes larger. The above adjustable design parameters should be carefully selected by trial and error so as to achieve the satisfactory control performance.*

**Remark 5.**  *Most of the existing output-constrained control schemes can only ensure the asymptotic stability for the closed-loop manipulator system. Alternatively, this work extends the output-constrained control scheme to the fixed-time convergence for the closed-loop system, and thus the joint tracking errors are not only confined to the prescribed time-varying bounds, but also converged within the fixed time. To the best of our knowledge, there are really limited existing control frameworks that have such performance under the same conditions.*

## 5. Experiments

To verify the correctness and feasibility of the proposed control framework, experiments were performed on the real-world RGM-based robotic manipulator system. The experiments consisted of three comparison studies, which verify the superiority of the BLF, the correctness and effectiveness of the RNN, and the fixed-time convergence of the proposed controller, respectively. The features and differences of each compared controller or case are illustrated in Table 1.

### 5.1. Experimental System Setup

**Figure 2:**
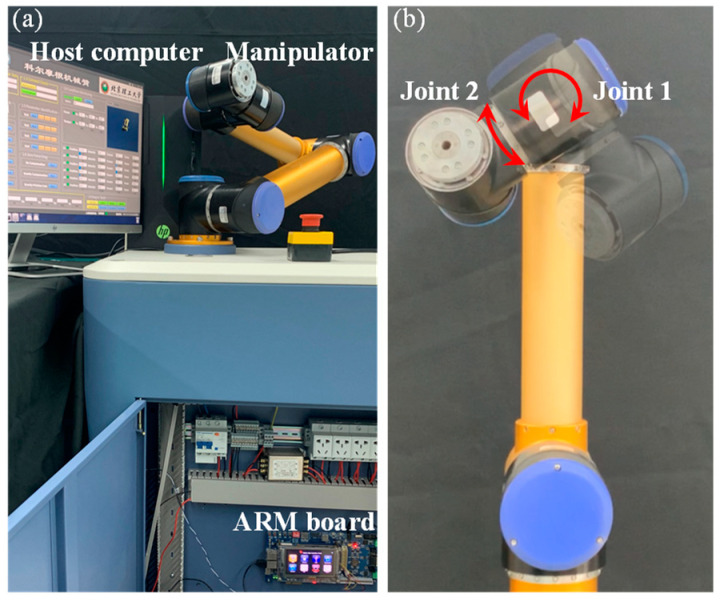
Experimental setup: (**a**) Experimental platform of the manipulator system; (**b**) Manipulator joints used in experiments, where the red arrows represent how the joints rotate.

The robotic system is a self-designed manipulator (see Figure 2a) based on GRM joints providing the torque control interface (RGM integration joints, Kollmorgen Co., Radford, VA, USA), and no prior knowledge of manipulator dynamics can serve this study. An ARM board with RT-LINUX system is responsible for running the proposed control algorithm written in the C language. Real-time joint angles, angular velocities, and control signals run on a CAN bus. The communication frequency of the CAN bus is 400 Hz. The update frequency of the online RNN approximator is also 400 Hz.

The control task is dynamic tracking in joint space, and two manipulator joints shown in Figure 2b are asked to track the desired trajectory given as $q_{d}={\left[sin\left(\pi t/4\right)-\pi /2,\pi /2cos\left(\pi /8\left(t+\pi /2\right)\right)sin\left(\pi /4\left(t+\pi /2\right)\right)\right]}^{T}$ rad, t≤ 32s. The initial states of the manipulator are given as $q\left(0\right)={\left[-78.54,84.27\right]}^{T}$ deg and $\dot{q}\left(0\right)={\left[0,0\right]}^{T}$. To ensure safe working of the manipulator, the maximum control torque is set as 40 Nm. Control parameters of the proposed FTVC (23) and FTRC (35) are empirically selected as $k_{1}=6$, $k_{2}=7$, $K_{1}=12$, $K_{2}=15$, α=99/101, β=103/101, Λ=10, and $\rho_{i}\left(t\right)=\left(0.4-0.01\right)exp\left(-0.1t\right)+0.01$, i=1,2. For the RNN approximator, ${\hat{U}}_{i}\left(0\right)=c$, ${\hat{W}}_{i}\left(0\right)=zeros\left(l,1\right)$, $\gamma_{1i}=100I_{l}$ $\gamma_{21}=6I_{l}$, $\gamma_{22}=5I_{l}$, $\eta_{1i}=0.0001$, and $\eta_{2i}=0.01$, i=1,2. Note that the control parameters of the proposed controller should be carefully set according to the instruction provided in Remark 4. The number of activation node l=13. The width of Gaussian basis function b=60 and c is uniformly spaced in $\left[-30,30\right]$. For the FOLPF (20), τ=0.01 and $\upsilon \left(0\right)={\left[-3.5146,-4.3930\right]}^{T}$. For the sake of convenience, the proposed fixed-time output-constrained RNN learning controller (35) is denoted as Controller 1.

### 5.2. Comparison Studies: Role of the BLF

First, to show the advantage of the BLF, the ablation scheme is utilized to compare the performance with and without the BLF. Specifically, referring to (29), the compared controller (Controller 2) is a simplified form of Controller 1, in which while the output constraint is removed, the DSC framework and RNN approximator are retained. To make a fair comparison, the RNN approximator parameters, FOLPF parameter, and the other control parameters of Controller 2 are chosen the same as those of Controller 1. The initial states of the manipulator and RNN approximator are also selected the same as those of Controller 1. Then, Controller 2 and its FTVC are designed as

(64) $$\left\{\begin{array}{l} u=-\Lambda e_{1}-K_{1}{\left\lfloor \left.e_{2}\right\rceil \right.}^{\alpha }-K_{2}{\left\lfloor \left.e_{2}\right\rceil \right.}^{\beta }-\frac{1}{2}e_{2}-{\hat{W}}^{T}\hat{\Phi }\left(p\right)\\ \overset{-}{\upsilon }=-k_{1}{\left\lfloor \left.e_{1}\right\rceil \right.}^{\alpha }-k_{2}{\left\lfloor \left.e_{1}\right\rceil \right.}^{\beta }-\frac{1}{2}e_{1}. \end{array}\right.$$

**Figure 3:**
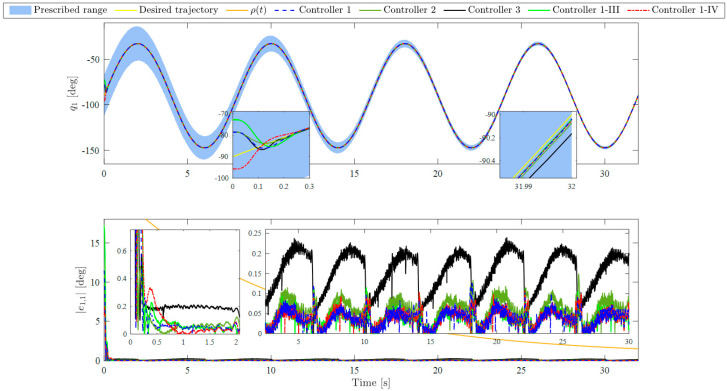
Trajectory tracking results and tracking errors of Joint 1 under different controllers and cases.

**Figure 4:**
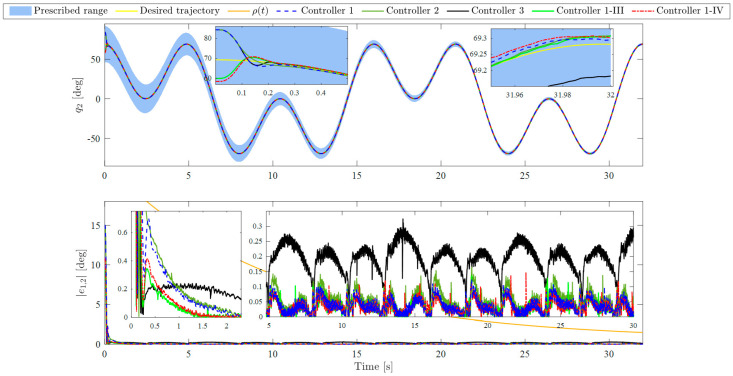
Trajectory tracking results and tracking errors of Joint 2 under different controllers and cases.

**Figure 5:**
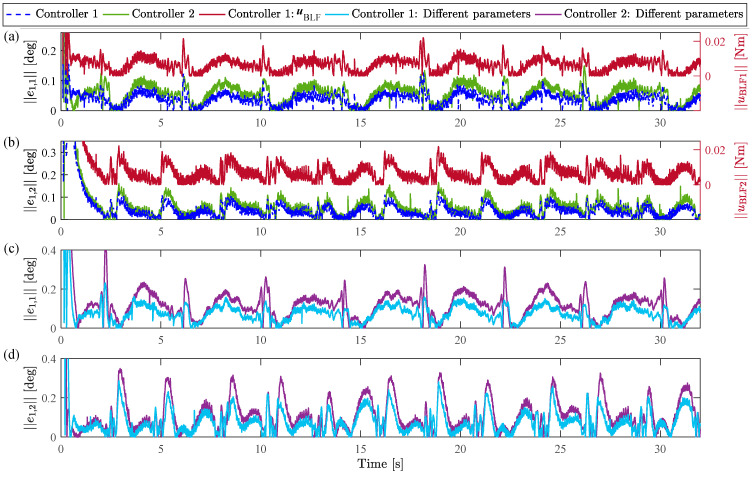
Comparisons of tracking error between Controller 1 and Controller 2: (**a**,**b**) are tracking errors and the corresponding control input $u_{BLF}$; (**c**,**d**) are tracking errors under smaller control parameters.

**Figure 6:**
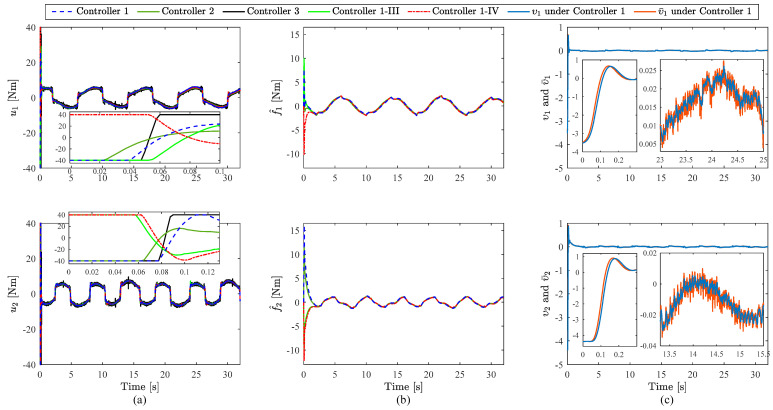
Comparison results: (**a**) Control torques; (**b**) Estimate values of f; (**c**) Comparison between the filtered virtual control signal and virtual control signal under Controller 1.

According to the FTVC in (64), the initial states of the FOLPF are selected as $\upsilon \left(0\right)={\left[-2.5014,-3.7944\right]}^{T}$. It should be noted that no external disturbance is acted on the system in this subsection, i.e., $\Delta D={\left[0,0\right]}^{T}$. The experimental results of Controllers 1 and 2 are presented in Figure 3, Figure 4, Figure 5 and Figure 6. Figure 3 and Figure 4 depict the joint tracking results and corresponding tracking errors of Joint 1 and Joint 2, respectively. Figure 6a shows the control torques. Figure 6b presents the RNN estimate values of f. Figure 6c represents the comparison between the filtered virtual control signal and virtual control signal under Controller 1. From Figure 3 and Figure 4, the dynamic tracking is successful under each controller even in the presence of unknown manipulator dynamics, and the real joint trajectories did not exceed the prescribed time-varying ranges under Controller 1. With the results of joint tracking errors, the tracking accuracy of Controller 1 is slightly higher than that of Controller 2 for both joints. In addition, RMSEs of $q_{1}$ and $q_{2}$ within the time interval 2–32 s are calculated and listed in Table 2. From Table 2, the RMSE of Controller 1 is obviously smaller than that of Controller 2 for each joint. In Figure 3 and Figure 4, while the difference in tracking performance between Controllers 1 and 2 is small, it does not negate the role of the BLF since the steady-state error is very small under the selected control parameters, which results in a small control input $u_{BLF}$ according to (18). Figure 5 shows more in-depth comparisons of the tracking performance between Controller 1 and Controller 2. Figure 5a,b show the tracking errors in Figure 3 and Figure 4 again, and present the corresponding control input $u_{BLF}$. It is easy to see that while $u_{BLF}$ increases as $e_{1}$ increases, its maximum value is only 0.02 Nm within the time interval 2–32 s, so the role of the BLF is small when the tracking error is very small. Figure 5c,d show the tracking errors when selecting smaller control parameters $k_{1}$, $k_{2}$, $K_{1}$, and $K_{2}$ for both controllers, i.e., $k_{1}=3$, $k_{2}=4$, $K_{1}=8$, and $K_{2}=9$. It can be observed that the tracking accuracy of Controller 1 is obviously higher than that of Controller 2 for both joints, which means the use of BLF can indeed reduce the error under the same conditions, and thus Controller 1 performs better than Controller 2. In addition, Figure 6a shows different control torques under Controllers 1 and 2 to illustrate the different operating states of the two controllers. Besides, the control torques for both controllers remain in the preset safe range for practical applications from the partially enlarged views of Figure 6a. To sum up, the above comparison results and analyses demonstrate the effectiveness and superiority of the BLF. In addition, under Controller 1, the filtered virtual control signal and the virtual control signal are almost identical all the time, and the virtual control signals can be well filtered through the FOLPF from the partially enlarged views of Figure 6c. Accordingly, the effectiveness of the DSC-based control framework can be verified. We can also observe from Figure 6b that the estimate values $\hat{f}$ of Controllers 1 and 2 are almost the same. These results are reasonable since the real lumped uncertainties f in the two experiments are the same. Therefore, the results in this subsection show that the estimate capability of the RNN is relatively stable. Further verification of the RNN will be studied in the next subsection.

### 5.3. Performance Verification of the RNN

To verify the correctness and effectiveness of the RNN approximator, the overall performance of Controller 1 is compared with that of the classical proportional-derivative (PD) controller (Controller 3) under external disturbances. The control parameters for Controller 1 are chosen the same as those in Section 5.1. Specifically, we introduce an external disturbance signal acting on Joint 1 at 16 s, which is given as

(65) $$\Delta D=\left\{\begin{array}{l} {\left[0,0\right]}^{T},t\leq 16\;s\\ {\left[-2sin\left(\pi t/4\right),0\right]}^{T}Nm,t>16\;s. \end{array}\right.$$

Controller 3 is designed as

(66) $$u=-K_{P}e_{1}-K_{D}{\dot{e}}_{1}$$

where $K_{P}>0$ and $K_{D}>0$ are control parameters of Controller 3 selected as $K_{P}=1500$ and $K_{D}=65$.

**Figure 7:**
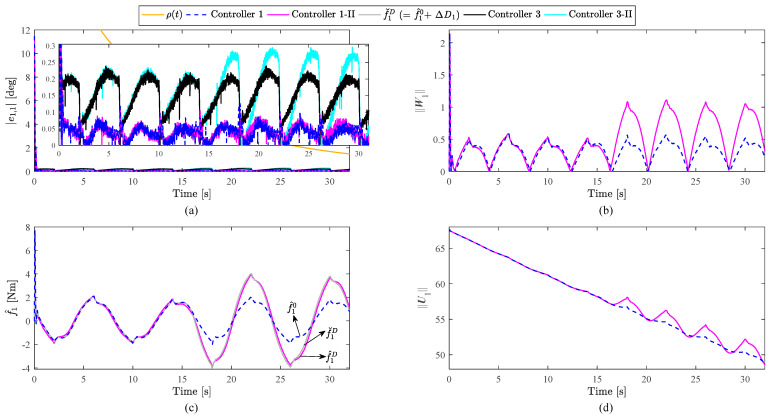
Comparisons of disturbance rejection: (**a**) Trajectory tracking errors of Joint 1; (**b**) Norms of forward neural weights for Joint 1; (**c**) Estimate values of $f_{1}$, where black arrows represent the correspondence between curves and variables; (**d**) Norms of recurrent neural weights for Joint 1.

The results of Controllers 1 and 3 under external disturbances are denoted as Controller 1-II and Controller 3-II, respectively. For convenience of comparisons, the results of Controller 3 without external disturbances are also given and shown in Figure 3, Figure 4, Figure 6a and Figure 7a. A video of experiment for Controller 1-II is provided in Supplementary Materials.

Comparisons of disturbance rejection between Controller 1 and Controller 3 are shown in Figure 7. Figure 7a depicts the trajectory tracking errors of Joint 1. Figure 7b,d show the norms of forward neural weights and recurrent neural weights for Joint 1, respectively. Figure 7c presents the estimate values of $f_{1}$. First, it is clearly seen from Figure 3, Figure 4 and Figure 7a that the tracking performance of Controller 3 is poorer than that of Controller 1 regardless of the presence of the external disturbance. From Figure 7a, the tracking error of Controller 3 obviously becomes larger after introducing the external disturbance, while the tracking error of Controller 1 changes little after introducing the external disturbance. In addition, RMSEs within the time interval 16–32 s are calculated and listed in Table 3 for comparisons of disturbance rejection. After introducing the disturbance, the RMSE of Controller 1 increased by 5.21% while the RMSE of Controller 3 increased by 18.93%. Second, we design a method to indirectly evaluate the estimate accuracy of the RNN since the real lumped uncertainties $f_{1}$ is unavailable. Note that the introduced sinusoidal external disturbance ${\Delta D}_{1}$ is unavailable for controllers while it is available for users; hence let ${\hat{f}}_{1}$ of Controller 1-II subtract ${\hat{f}}_{1}$ of Controller 1 and denote the difference as ${\Delta \hat{D}}_{1}$. If ${\Delta \hat{D}}_{1}$ is close to the real external disturbance ${\Delta D}_{1}$, the lumped uncertainties are well estimated and the correctness of the RNN approximator can be guaranteed. For convenience of expression, ${\hat{f}}_{1}$ of Controller 1-II and ${\hat{f}}_{1}$ of Controller 1 are denoted as ${\hat{f}}_{1}^{D}$ and ${\hat{f}}_{1}^{0}$, respectively. For better graphical expression, we do not calculate ${\Delta \hat{D}}_{1}$, but define ${\overset{˘}{f}}_{1}^{D}≝{\hat{f}}_{1}^{0}+{\Delta D}_{1}$, which is represented by a gray solid line in Figure 7c. In this way, the estimated performance of the RNN can be evaluated by comparing the curves of ${\overset{˘}{f}}_{1}^{D}$ and ${\hat{f}}_{1}^{D}$. The results of Figure 7c show that the RNN is accurate in estimating the external disturbance since ${\hat{f}}_{1}^{D}$ and ${\overset{˘}{f}}_{1}^{D}$ are very close. To sum up, the above results of two aspects (tracking error and estimate accuracy) demonstrate the correctness and effectiveness of the RNN approximator, the strong capability of disturbance rejection for the proposed controller, as well as the better overall performance for the proposed controller compared with the PD controller. From Figure 7b,d, the forward neural weights and recurrent neural weights are constantly tuned to cope with the time-varying lumped uncertainties in different situations.

**Remark 6.**  *In the RNN, while the network is becoming more complex due to the addition of recurrent loops, it can be adopted as long as the computational frequency of hardware for NN can meet the requirement of the control frequency. According to the existing literature, there is no online estimate that the recurrent loop and NN dynamics in a robust form are both considered, and the excellent overall performance for the closed-loop system is achieved. Thus, the robust online RNN approximator is first developed for tracking control*.

### 5.4. Fixed-Time Convergence Verification

To verify the fixed-time convergence of Controller 1, we conduct two cases containing different initial states of the manipulator. Two cases are denoted as Controller 1-III and Controller 1-IV, respectively. Considering the restriction on feasibility condition for Tan-BLF, $q\left(0\right)$ should be selected to stay within the prescribed ranges. In Controller 1-III, $q\left(0\right)={\left[-72.81,60\right]}^{T}$ deg, $\dot{q}\left(0\right)={\left[0,0\right]}^{T}$, and $\upsilon \left(0\right)=\left[-3.5247,\right.{\left.3.4959\right]}^{T}$. In Controller 1-IV, $q\left(0\right)={\left[-95.73,58.49\right]}^{T}$ deg, $\dot{q}\left(0\right)={\left[0,0\right]}^{T}$, and $\upsilon \left(0\right)={\left[1.9251,3.9247\right]}^{T}$. In this section, the control parameters for both cases are chosen the same as those in Section 5.1 and $\Delta D={\left[0,0\right]}^{T}$.

The experimental results of Controllers 1-III and 1-IV are provided in Figure 3, Figure 4 and Figure 6. It can be observed that the real joint trajectories of the two cases did not exceed the prescribed time-varying ranges, and the settling time and steady-state errors of Controllers 1, 1-III, and 1-IV are almost the same even with different initial conditions of the closed-loop system. These results indicate that Controller 1 exhibits the fixed-time convergence ability, whose settling time is bounded and independent of the initial system states. Additionally, from Figure 6b, the estimate $\hat{f}$ values of Controllers 1, 1-III, and 1-IV are almost the same; hence the effectiveness of the RNN approximator is verified again, and these results demonstrate the stable estimate capability of the RNN approximator.

## 6. Conclusions

In this paper, the authors were devoted to designing a fixed-time RNN learning control framework using the Tan-BLF for the dynamic tracking of manipulators. The experimental results show that the proposed RNN method not only possesses the competence as an online approximator of uncertain systems, even in the presence of unknown manipulator dynamics and external disturbances, but also achieves better anti-disturbance performance compared with the PD controller. Such performance demonstrates that the designed NN makes the controller have significant online adaptable ability and stable estimate capability. In addition, the proposed control framework not only guarantees the joint tracking errors converge to the small neighborhoods about the origin in fixed time, but it also always preserves the joint angles within the prescribed ranges and thus improves the tracking accuracy. It is the first time that the dynamic tracking control problem of a real-world manipulator with unknown dynamics is studied based on an RNN learning approach with the consideration of a time-varying constraint method. In this study, only two joints of the manipulator are used to verify the effectiveness and superiority of the proposed algorithm, and we will apply this algorithm to all joints of the manipulator to solve the task space control problem in future research.

## Figures and Tables

**Table 1:** Features and differences of each compared controller or case.

	DSC	PD	BLF	RNN	Disturbance ΔD	Initial Position $q\left(0\right)$/[deg]
Controller 1	✓	—	✓	✓	—	${\left[-78.54,84.27\right]}^{T}$
Controller 2	✓	—	—	✓	—	${\left[-78.54,84.27\right]}^{T}$
Controller 3	—	✓	—	—	—	${\left[-78.54,84.27\right]}^{T}$
Controller 1-II	✓	—	✓	✓	✓	${\left[-78.54,84.27\right]}^{T}$
Controller 3-II	—	✓	—	—	✓	${\left[-78.54,84.27\right]}^{T}$
Controller 1-III	✓	—	✓	✓	—	${\left[-72.81,60\right]}^{T}$
Controller 1-IV	✓	—	✓	✓	—	${\left[-95.73,58.49\right]}^{T}$

Notations: “✓” refers to the use of the algorithm. “—” refers to not using the algorithm.

**Table 2:** Comparisons of RMSE between Controller 1 and Controller 2.

RMSE within 2–32 s [deg]	Controller 1	Controller 2
$q_{1}$	0.0426	0.0639
$q_{2}$	0.0432	0.0601

**Table 3:** Comparisons of disturbance rejection between Controller 1 and Controller 3.

$\mathrm{RMSE}\;\mathrm{of}\;q_{1}$ within 16–32 s [deg]	Controller 1	Controller 3
Without disturbance	0.0461	0.1675
With disturbance	0.0485	0.1992
Rate of change	5.21%↑	18.93%↑

## Data Availability

Not applicable.

## Supplementary Materials

The following supporting information can be downloaded at: https://www.mdpi.com/article/10.3390/s23125614/s1, A video of experiment is provided in supplementary materials.
